# Molecular stripping, targets and decoys as modulators of oscillations in the NF-κB/IκBα/DNA genetic network

**DOI:** 10.1098/rsif.2016.0606

**Published:** 2016-09

**Authors:** Zhipeng Wang, Davit A. Potoyan, Peter G. Wolynes

**Affiliations:** 1 Center for Theoretical Biological Physics, Rice University, Houston, TX 77005, USA; 2 Department of Chemistry, Rice University, Houston, TX 77005, USA; 3 Department of Physics and Astronomy, Rice University, Houston, TX 77005, USA

**Keywords:** NF-κB, molecular stripping, genetic oscillations, systems biology, synthetic biology

## Abstract

Eukaryotic transcription factors in the NF-κB family are central components of an extensive genetic network that activates cellular responses to inflammation and to a host of other external stressors. This network consists of feedback loops that involve the inhibitor IκBα, numerous downstream functional targets, and still more numerous binding sites that do not appear to be directly functional. Under steady stimulation, the regulatory network of NF-κB becomes oscillatory, and temporal patterns of NF-κB pulses appear to govern the patterns of downstream gene expression needed for immune response. Understanding how the information from external stress passes to oscillatory signals and is then ultimately relayed to gene expression is a general issue in systems biology. Recently, *in vitro* kinetic experiments as well as molecular simulations suggest that active stripping of NF-κB by IκBα from its binding sites can modify the traditional systems biology view of NF-κB/IκBα gene circuits. In this work, we revise the commonly adopted minimal model of the NF-κB regulatory network to account for the presence of the large number of binding sites for NF-κB along with dissociation from these sites that may proceed either by passive unbinding or by active molecular stripping. We identify regimes where the kinetics of target and decoy unbinding and molecular stripping enter a dynamic tug of war that may either compensate each other or amplify nuclear NF-κB activity, leading to distinct oscillatory patterns. Our finding that decoys and stripping play a key role in shaping the NF-κB oscillations suggests strategies to control NF-κB responses by introducing artificial decoys therapeutically.

## 1. Introduction

**Figure 1.**
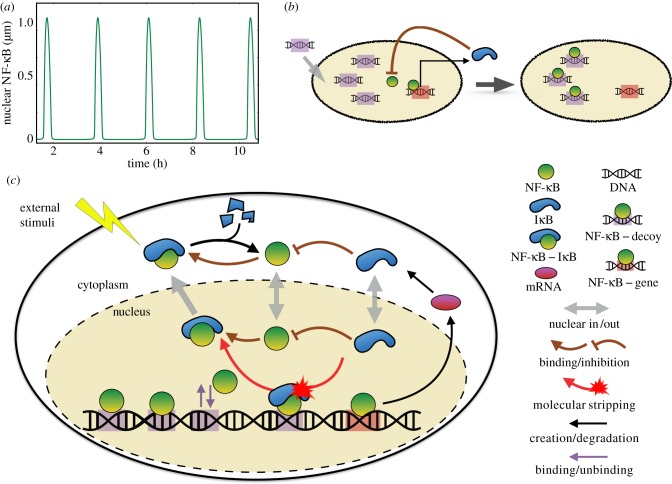
(*a*) Nuclear NF-κB oscillations of the minimal deterministic model used in this work. (*b*) Simplified illustration of the idea of using exogenous decoys for suppressing NF-κB activity via sequestration (*c*). Detailed schematic of the 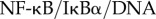 minimal circuit. Shown are the key steps and molecules. Under external stimuli, the 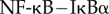 complex is marked for selective degradation of the IκBα. Subsequently, the freed NF-κB translocates into nucleus, binding to unoccupied DNA sites including both decoys (purple) and the promoter of IκBα gene (red). The NF-κB binding to the promoter, initiates transcription of mRNA that, in turn, leads to the synthesis of IκBα in the cytoplasm. The free IκBα inhibits the activity of nuclear NF-κB by converting it back to 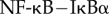. The inhibition involves binding of IκBα to NF-κB (brown arrows) as well as stripping from the DNA-bound complexes in the nucleus (red arrow).

The regulatory network based on the transcription factor NF-κB has a broad range of influence in eukaryotic cells, which includes orchestrating immune response to inflammation, apoptosis, proliferation, differentiation and many more activities [1]. The nuclear factor of kappa B denotes a class of structurally related dimeric proteins where in our study the term NF-κB refers specifically to the p50–p65 heterodimer, which is the predominant complex in most cells. Remarkably, the central part of the NF-κB network contains only a few genes whose interplay leads to an oscillatory feedback cycle (figure 1*a,c*). The key actor here is the gene coding for the inhibitor of NF-κB: IκBα. Again, IκB denotes a class of inhibitors with IκBα being the most dominant inhibitor of NF-κB. This IκBα feedback leads to the oscillations that have been observed both in single cells [2,3] and in populations of cells [4]. In addition to the central feedback core, there are huge numbers of NF-κB binding sites that are sprinkled widely across the genome [5–7]. Some of these sites are actual genes encoding proteins for regulating signalling downstream, but the large majority seem to act only in some fashion as decoys and are not known to have specific functions. The latest Chip-seq experiments suggest there are at least approximately 2–3 × 10^4^ [5] sites populated by the NF-κB but only approximately 500 of these are known to be protein coding genes [8,9]. It is estimated there are 10^5^ [10] copies of the NF-κB in a typical eukaryotic cell, so the large number of binding sites is able to sequester a significant fraction of the nominally free NF-κB. Whether the large number of decoys is an ‘accident of nature’ related to the statistics of binding to short DNA signals or a feature evolved by natural selection is unclear.

The recent discovery of molecular stripping of NF-κB from DNA binding sites by IκBα [11,12] suggests that in addition to passive dissociation of NF-κB from binding sites, as in the classical systems biology model of the NF-κB/IκBα switch, transcription factors can be stripped actively from either a decoy or a gene promoter site dependent on concentration of IκBα [12]. This dependence changes the way in which the NF-κB/IκBα circuits sets up its oscillations (figure 1*c*). This novel mechanism opens up new ways of modulating the time-dependent signals that are being broadcast by the circuit: by varying the number of binding sites, by making genetic and epigenetic changes of DNA sequence, by mutating transcription factors and by modifying the kinetics of molecular stripping events [13].

Here, we task ourselves with exploring how these molecular kinetic events change the systems-level behaviour of the NF-κB network under steady stimulation where the system displays self-sustained oscillations. Throughout the work, we treat all the alternate binding sites as being decoys, i.e. simply additional binding sites for the NF-κB without regard to their nature, be they functional targets for activating signals or purely non-functional decoys. We therefore assume that the signals arising downstream from the functional targets and any of the decoys are sufficiently unentangled from the main feedback loop so as to not affect the timescales of oscillations. Nevertheless, we show that decoys do influence in non-trivial ways the overall system properties by directly acting as sinks for the NF-κB which alters the steady-state balance of molecules in the feedback cycle. Specifically, we show how the amplitude and the frequency of oscillation as well as the nuclear to cytoplasmic ratio of NF-κB are controlled via decoy sites and molecular stripping. We show this by computationally varying the number of decoys, and varying the timescale of unbinding from decoys via passive dissociation and molecular stripping events in a quantitative deterministic systems biology model of the circuit.

Because several afflictions, including cancer, arthritis, chronic inflammation, asthma, neurodegenerative diseases and heart disease have been found to be caused by misregulation of active NF-κB, countermeasures to aberrant gene expression, using artificially designed therapeutic DNA decoys have been developed with the aim of inhibiting transcription of NF-κB [14,15]. These therapeutic decoys are exogenous short consensus binding sequences designed to outcompete the natural, genomic DNA targets for binding to transcription factors (figure 1*b*). The simple and powerful idea of silencing undesirable gene activities by sequestering the corresponding transcription factors should be broadly also applicable to other eukaryotic master regulators [16]. Exploring the use of therapeutic decoys to suppress the NF-κB activities has been pursued by many research groups and has already shown much promise [17,18].

The development of caged [19] and catch and release [20] NF-κB decoys capable of being turned on and off via photochemical activation by UV light offers the possibility for full spatio-temporal control of NF-κB regulation using exogenous decoys. The realization of the full medical potential of such decoys, however, is currently stymied by many challenges [16] that could possibly be overcome by deepening our understanding of the dynamics of NF-κB regulation simultaneously at the molecular and at systems levels. Exploring the role of molecular stripping in determining the influence of decoys is a step forward towards this understanding.

## 2. Mathematical models of the NF-κB regulatory network

Many techniques are available for modelling genetic networks [21]. At one end of the spectrum of modelling, tools are relatively coarse logic-based models that assume minimal knowledge about the detailed interactions of molecules. At the other end, models are based on a stochastic description of reactions and physical transport in a structured cell [22,23]. In this work, we choose a mid-level description for in depth exploration of the space of kinetic regimes of our revised minimal model of the NF-κB network (figure 1*c*), incorporating molecular stripping and decoys. We adopt a first principle chemical kinetic model in its deterministic formulation that includes specific effects of transcription factor binding to DNA sites.

The regulatory network of NF-κB has already been the object of extensive mathematical modelling, studies using both deterministic and stochastic models of oscillations [24,25].

In the main, the phenomenology of these models can be traced back to the work of Hoffmann *et al.* [4], who developed an extensive network of more than 20 reactions whose parameters were fitted to experiments on cellular populations. Their modelling philosophy used all the biochemical information pertaining to NF-κB and IκBα pathways that was available at the time the model was formulated. Variations of this early model have been able to account for the oscillatory patterns that are also seen in single-cell experiments. These studies show that a sufficient condition for oscillations is the existence of a core feedback cycle with appropriate timescales. Nevertheless, we must note that the successful fitting of experimental data with a particular systems-level model does not rule out the significance of other biochemical processes that also can give appropriate feedback. Constructing minimal models is nevertheless essential for building hypothesis about the network dynamics without overfitting to the experiments, which can be sensitive to detailed laboratory protocols and initial conditions [26]. Therefore, minimalist models have become increasingly important for understanding the NF-κB network [27–30]. The present minimal model, which is based entirely on an explicit mass action treatment of the kinetics of elementary reactive events that accounts for both the binding states of gene promoter and decoy sites. The model incorporates bimolecular molecular stripping of NF-κB from bound sites by IκBα in addition to the spontaneous unimolecular dissociation used in the earlier models. The names of the molecular species involved, their associated reactions and kinetic coefficients are shown in tables 1 and 2. The corresponding set of ordinary differential equations that constitutes our model is presented below.

**Table 1.** Chemical reactions for IκBα/NF-κB regulatory circuit. The parameters of the feedback cycle originate from the work of Hoffmann *et al.* [4], whereas the ranges of values for specific binding/unbinding rates come from binding microarray data [31] and *in vitro* kinetic measurements [32,11].

reactions	rate coeff.	values
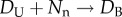	*k*_don_	[0–10] µM^−1^ min^−1^
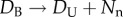	*k*_doff_	[0–10] min^−1^
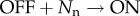	*k*_on_	[0–10] µM^−1^ min^−1^
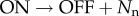	*k*_off_	[0–10] min^−1^
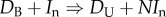	*k*_s_	[0–100] µM^−1^ min^−1^
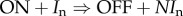	*k*_s_	[0–100] µM^−1^ min^−1^
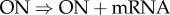	*k*_tr_	1.03 µM^−1^ min^−1^
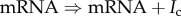	*k*_tl_	0.2448 min^−1^
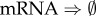	*k*_d_	0.017 min^−1^
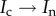	*k*_in_	0.018 min^−1^
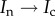	*k*_out_	0.012 min^−1^
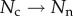	*k*_Nin_	5.4 min^−1^
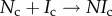	*k_f_*	30 µM^−1^ min^−1^
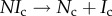	*k*_b_	0.03 min^−1^
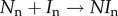	*k*_fn_	30 µM^−1^ min^−1^
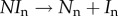	*k*_bn_	0.03 min^−1^
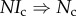	*α*	0.55 min^−1^
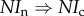	*k*_NIout_	0.83 min^−1^

**Table 2.** Names of species and their numbers.

abbreviation	full name
*D*_B_	bound decoy site
*D*_U_	unbound decoy site
ON	active gene state
OFF	inactive gene state
*I*_n_	nuclear IκBα
*I*_c_	cytoplasmic IκBα
*N*_n_	nuclear NF-κB
*N*_c_	cytoplasmic NF-κB
*NI*_n_	nuclear 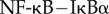 complex
*NI*_c_	cytoplasmic 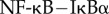 complex
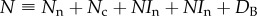	total number of NF-κB: 10^5^
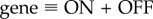	total number of *genes*: 1
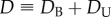	total number of *decoys*: 0–10^5^

Because there is a single IκBα promoter and *D* number of copies of decoys (which are neither synthesized nor destroyed), stoichiometry requires that [ON] + [OFF] = 1 and [*D*_B_] + [*D*_U_] = *D*. The total number of NF-κB molecules is another quantity that remains constant in the model and is set to a typical value for eukaryotes, which is about approximately 10^5^. We adopt the units of micromolar concentration for all the species. Using the appropriate total volume for eukaryotic cells, we have that 1 µM corresponds to there being 10^5^ NF-κB molecules in the cell.2.1
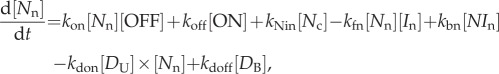
2.2
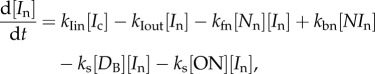
2.3
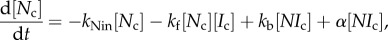
2.4
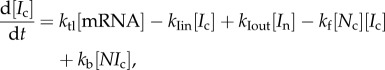
2.5
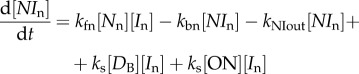
2.6
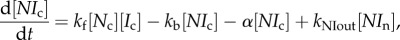
2.7
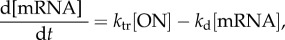
2.8
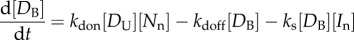
2.9
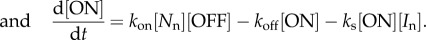


This set of ordinary differential equation (ODE) was solved using the integrator of real-valued variable-coefficient ODE solver, with fixed-leading-coefficient as implemented in Scipy library of python2.7. The parameters were scanned on a fine grid within the ranges specified in table 1. Oscillatory dynamics was propagated for 3000 min discarding the first 500 min to eliminate any possible biases owing to initial conditions.

## 3. Results

We systematically scanned all the rate processes related to binding/unbinding to decoys and to the promoter by varying the unbinding rates from the decoys, *k*_doff_ (=0.1 · *k*_don_) and the gene promoter, *k*_off_ (0.1 · *k*_on_), the total number of decoys (*D* = *D*_B_ + *D*_U_) and also the molecular stripping rate, *k*_s_. Most of these parameters have been measured in studies employing real-time *in vitro* DNA binding kinetic experiments [32,11] and genomewide microarray data [31] conducted on the p50p65 heterodimer of NF-κB and fall within the range of values employed by us. The model takes the binding affinities and stripping rates to be the same for all of the decoys, although these doubtless take on a range of values for different sites as is seen in the experimental measurements, using different DNA motifs [31]. Because our objective is to investigate the relationship between oscillatory behaviour and the molecular properties of the decoys and the gene promoter, we focus on the amplitude and period of the oscillations, the nuclear-to-cytoplasmic ratio and the mean occupancy of the bound decoys.

**Figure 2.**
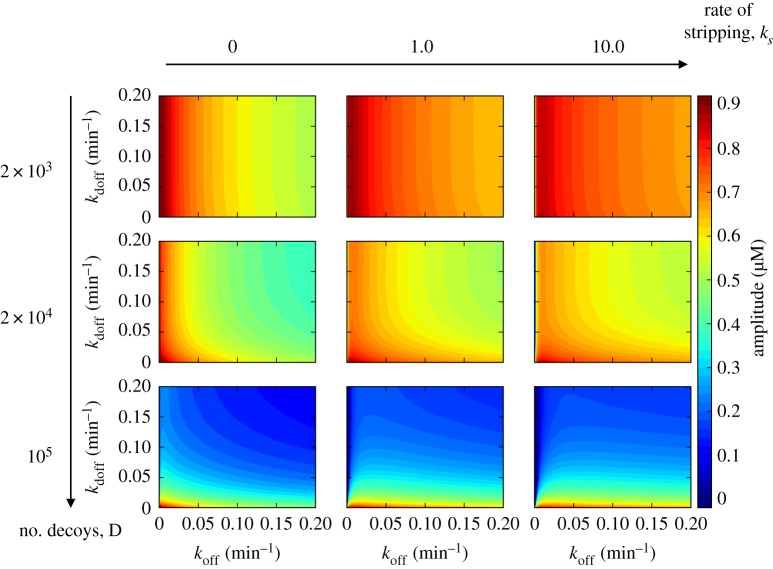
The amplitude of nuclear NF-κB oscillations as a function of dissociation timescales from decoys *k*_doff_ and gene promoter *k*_off_ under regimes set by different decoy numbers (columns) and molecular stripping rates (rows).

Figure 2 gives a broad overview of how the rates associated with the gene promoter and decoy binding influence the steady-state amplitude of nuclear NF-κB oscillations. Figure 2 highlights several distinct kinetic regimes. First, we note that in order for these unbinding rates to have any influence on oscillations their timescales need to be comparable to the timescale of the oscillatory period *τ*_off_ ∼ *τ*_doff_ ∼ *T*. The feedback loops produce oscillations in the range of hours, so we scan the values of rate coefficients, giving timescales ranging from several minutes to hours.

The most prominent feature of figure 2 is the reduction of the oscillatory amplitude by increasing the number of decoys. This pattern of reduction holds true, regardless of the other rates in the network. The addition of more decoy binding sites to the nucleus simply binds up more nominally free NF-κB hence reducing its concentration. The timescales of binding and unbinding for decoys, however, matters, because ‘faster decoys’ reduce the amplitude of free NF-κB change more effectively. If the free NF-κB were in equilibrium with the decoys, then its amplitude would not change when varying the individual rates so long as the binding free energy reflected in the equilibrium constant is held fixed *k*_doff_/*k*_don_ = 0.1 µM. The free nuclear NF-κB, however, is clearly not at equilibrium because it oscillates with sharp spikes of duration *t*_sp_ which are each followed by a longer period of near zero concentration, 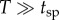. Thus, for the decoys to be able to reduce the amplitude of free NF-κB variation, they have to bind them within the narrow spiking window, which is why ‘faster decoys' are more effective in the limit of 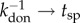. This confirms the intuition behind the idea of using the decoys for therapy while also showing that one can design more effective decoys by tuning their binding/unbinding rates.

Second, we see that in the low decoy number regime (e.g. *D* ∼ 10^3^) both the state amplitude (figure 2) and the period of oscillations (electronic supplementary material, figure S1) are sensitive to rates of promoter unbinding while they are not affected by varying the unbinding rates of decoys. In this regime, the number of decoys 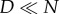 is too small to have any notable influence on the oscillatory dynamics NF-κB. On the other hand, the rate of unbinding from a single promoter site affects the oscillations significantly by virtue of the promoter being part of the core feedback cycle. Slower unbinding from the promoter leads to longer delays and larger amplitudes of oscillation. With an increasing number of decoys, however, one sees the binding kinetics of decoys becomes more influential (figure 2). When the decoy numbers approach the number of total NF-κB molecules (e.g. *D* ∼ 10^5^), the oscillatory dynamics becomes largely governed by the rates of binding to decoys.

**Figure 3.**
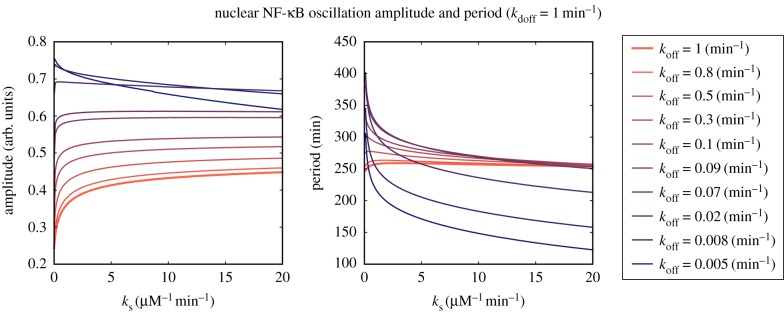
The amplitude and period of nuclear NF-κB oscillations plotted as a function of molecular stripping rates for different gene promoter dissociation *k*_off_ rates. The total number of decoys is fixed at *D* = 2 × 10^4^ with a dissociation rate from decoys set at: *k*_doff_ = 1 min^−1.^

Molecular stripping appears to naturally counter the effect of decoys by leading to a higher amplitude of NF-κB oscillations. The exception is when the unbinding of the NF-κB promoter is so slow as to become the rate limiting step of the feedback cycle. In figure 3, we analyse the latter situation in greater detail.

With respect to the susceptibility of changing NF-κB oscillations to changing the rate of molecular stripping, there are two distinct regimes of slow and fast gene promoter state changes. These regimes are revealed by plotting the steady-state amplitude and the periods as functions of the molecular stripping rate for different gene unbinding rates. We see that for more rapid gene state changes (*k*_off_ > 0.02 min^−1^) the oscillatory amplitude undergoes a modest increase with stripping. This change mostly occurs at the expense of depleting the number of bound decoys (see the electronic supplementary material), whereas the period of oscillations is largely unaffected. On the other hand, as the gene state dynamics becomes slower (*k*_off_ < 0.02 min^−1^), we see the reversal of the previous trend. Now, instead there is a drop in the oscillation amplitude with increasing stripping rates, whereas the period is also altered significantly. This latter regime of slow promoter binding/unbinding is where oscillatory dynamics becomes most susceptible to changes of molecular stripping rates. This is so because in the limit of very slow promoter unbinding, unbinding itself becomes the rate limiting step in the feedback cycle hence the rate of molecular stripping now strongly affects not only the concentrations of bound and free decoys, but also affects the state of the promoter by effectively increasing its unbinding rate (electronic supplementary material). Starting from the slow promoter and increasing the rate of molecular stripping we see that eventually promoter unbinding crosses a point after which molecular stripping is no longer a rate limiting step (figure 3).

**Figure 4.**
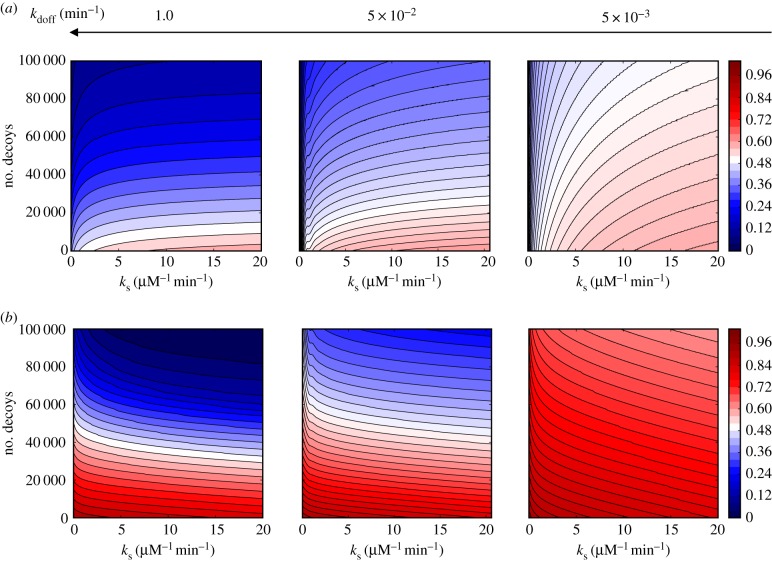
The amplitude of the nuclear NF-κB oscillations plotted as a function of the number of decoys and molecular stripping rate for the regimes of (*a*) fast, 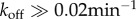 and (*b*) slow IκB promoter state change, 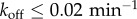.

To see whether changing the number of decoys changes the susceptibility of oscillations to molecular stripping, we scan over a wide range of values of decoy numbers and decoy unbinding rates in each of the identified regimes with slow and fast promoter unbinding. As figure 4 shows, the previously identified regimes (figure 3) are qualitatively the same for a wide range of decoy numbers. Quantitatively, however, in each fast/slow promoter regime the decoy unbinding rates change the degree to which the oscillation amplitude and frequency are susceptible to changes in molecular stripping rate and decoy numbers. As discussed previously (figure 2), the ‘faster decoys’ generally lead to smaller amplitude oscillations. Figure 4 now shows that depending on the unbinding rate from decoys and promoter there can be either cooperation where increasing the number of decoys and stripping both favour the reduction of the amplitude or lead to a tug of war where molecular stripping drives the system to higher amplitudes (figure 4).

Looking at the changes of period within the slow and fast promoter regimes (electronic supplementary material, figure S2), we see that the addition of decoys can strongly modulate the timing of oscillations in both of these regimes. Decoys do this by competitively reducing the binding to promoter by sequestering free NF-κB in the nucleus. This sequestration leads to delays in the feedback cycle resulting in longer periods. Molecular stripping counters these decoy induced delays by accelerating the switching in the slow promoter regime. In the fast promoter regime, the effect of stripping is negligible, because the rate limiting step of the feedback cycle is no longer the unbinding from the promoter. All of these relationships between decoys and stripping have potentially important implications for the therapeutic application of decoys. We see that there is more than one way of repressing the oscillatory activity of the nuclear NF-κB.

**Figure 5.**
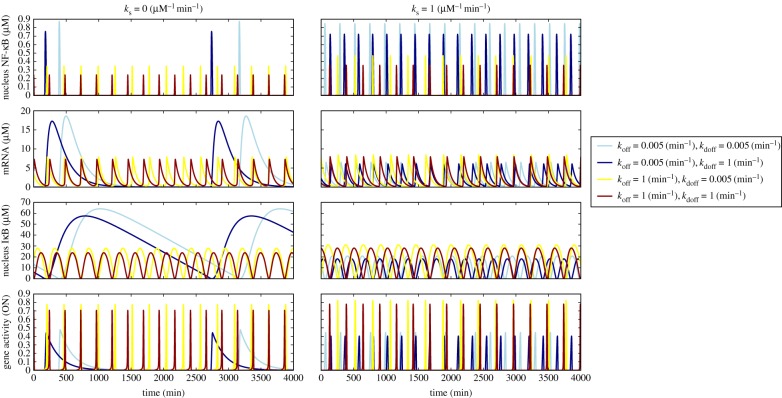
Shown are oscillatory trajectories of nuclear NF-κB, mRNA, nuclear IκBα and promoter state for the network without (left column) and with molecular stripping with respective rates set at *k*_s_ = 0 µM^−1^ min^−1^ and *k*_s_ = 1 µM^−1^ min^−1^. Molecular stripping is seen as restoring coherence of oscillations for all of the species regardless of unbinding rates on binding sites.

Finally, we examine how the disparity in timescales of unbinding from decoys and gene promoter affects the oscillations of different species and how oscillations are affected by the molecular stripping-like effects (figure 5). We looked at four possible scenarios where one has a slow promoter (blue lines) or a fast promoter (red lines) with either slow or fast decoys. First, we analyse the scenario without molecular stripping (left column of figure 5). We see that it is possible to obtain oscillations that are not coherent between different cells having different decoy and promoter unbinding rates even at the deterministic level. The disparity in oscillation frequencies tends to be greater in the case of a slow promoter and different rates of unbinding from decoys. The disparity is likely to be even more pronounced once one accounts for finite molecule number effects. Now when we look at the scenario with molecular stripping acting on bound sites, we see that this time all of the coherence of oscillations is restored with the period being nearly the same for all combinations of promoter and decoy unbinding rates. This shows one possible global role of molecular stripping which reduces variations of rates in the eukaryotic circuits caused by molecular disparities for spontaneous release from a wide range of binding sites. There are more nuanced changes that molecular stripping introduces into the system-level dynamics. These are best seen by considering limit cycles in the space of concentrations of different species (electronic supplementary material, figures S3–S6). For instance, limit cycle plots in the space of decoy bound and free nuclear NF-κB show that increasing molecular stripping rates tend to produce increasingly spiked pulses of decoy bound/free states (electronic supplementary material, figures S3). The degree of spikiness of oscillations has been argued previously [27] to be important for inducing sensitivity responses needed for differentially regulating downstream genes.

## 4. Discussion

This study shows that myriad of DNA binding sites on the genome, termed as decoy sites should be viewed as important species in the NF-κB network on an equal footing alongside the biochemical species which are part of the core feedback cycle of regulation. The oscillatory dynamics of all of the species is found to depend on the numbers and timescales of dissociation from these decoys. The main mode of influence of decoys is via sequestration of free nuclear NF-κB that leads to modulation of the NF-κB-activated oscillatory feedback. We find the influence of decoys on dynamics of the network grows as timescales of binding and unbinding approach NF-κB pulse duration. This offers ways to engineer exogenous decoys for modulating NF-κB activities. The recently discovered molecular stripping process [11,12], in turn, is able to influence the crosstalk between decoys and reactions in feedback cycles. Our results show that molecular stripping could be a way of enforcing coherent oscillations across population of cells, overcoming the inherent disparity of dissociation rates from the binding sites on individual genomes.

The ideas presented here in the context of a natural system can also aid in the modular design of synthetic oscillators or other complex engineered genetic circuits. An obstacle to modular design has been the problem of retroactivity [33,34], where decoy sites of one module sequester the transcription factors from another one when the modules are connected thereby making the overall design less efficient and at times even unpredictable. That the sequestration of transcription factors by decoys can lead to qualitative changes in transcriptional response has been demonstrated experimentally using a synthetic system in budding yeast [35]. In the work of Jayanthi *et al.* [36], it was shown that one can deal with retroactivity in a simple manner by tuning the periods and amplitudes of model synthetic oscillators by adding more copies of the operators. The related work by Karapetyan *et al.* [37] shows that adding a small number of operators can increase the coherence of generic activator and repressor oscillators. While adding operators can be a very effective means for reducing the dichotomous noise and directly changing the delay in the feedback loops, this multiplication of resources may not be effective for circuits that have a large number of targets as is the case of NF-κB. Apparently, evolution has chosen an alternative, molecular stripping, in the case of the NF-κB system. The dynamic properties of the network are easier to tune via molecular stripping rates that can globally enforce coherence of oscillations at a desired amplitude and period. Nature's way of solving the retroactivity problem by molecular stripping may also be a valuable addition to the toolbox of synthetic biologists.

In view of the large interest in developing anti-inflammatory and anti-cancer therapies which aim to suppress NF-κB pathways [38], this work offers us some promising alternatives to pure decoy-based therapy. Because IκBα is seen as a potentiator of the adverse effects of chemotherapy and radiation therapy [38], a more powerful strategy may be to design drugs binding to IκBα as an adjuvant to inhibit the molecular stripping before injecting decoys that would allow them to more effectively counter the aberrant activities of NF-κB.

While we have focused on a specific model system, the NF-κB network, a similar set of considerations probably apply to many other eukaryotic circuits where transcription factors usually bind to large number of sites on chromosomes. In this regard, we note that recently molecular stripping-like phenomena have been observed by a number of research groups by probing mechanistic aspects and kinetics of transcription factor dissociation in both eukaryotic and prokaryotic cells [39–43]. The full extent of the generality and functional implications of such mechanisms in systems biology of gene regulation are yet to be understood.

This work has addressed the systems biology implications of molecular stripping in the context of gene regulatory network of 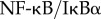. The theoretical framework of this work can be seen as an extension to the oscillating non-equilibrium dynamic situation of the physical chemistry of competitive binding of ligands to independent sites that are usually encountered in an equilibrium context. Similar considerations might be applicable to post-transcriptional regulation where instead of DNA decoys one must consider decoys based on RNA [44]. In analogy to transcription factors binding to DNA, there are proteins that bind to RNA thereby regulating the activities of the associated genes. The untranslated regions of mRNAs contain myriad binding sites for the post-transcriptional regulators, hence the analogy with this work is rather striking. Could it be that the workings of complicated gene regulatory networks in eukaryotes are most easily rationalized when viewed in the light of physico-chemical ideas resting on competitive binding of proteins to decoys and active dissociations from them? This study shows the usefulness of viewing the regulation of NF-κB as an interplay between cellular-level reactions in core feedback cycle and molecular association, and dissociation events on genomic sites. We hope that such an approach will allow integrating the structural insights from studies of 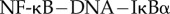 interactions on molecular level with the systems-level relationships learned from cell biology studies into a more coherent picture of gene regulation.

## Supplementary Material

Supplementary_fixed.pdf
