# Supplementary material for: Molecular stripping, targets and decoys as modulators of oscillations in the NF-κB/IκBα/DNA genetic network
Source: J R Soc Interface. 2016 Sep;13(122):20160606. doi: 10.1098/rsif.2016.0606 (PMC5046959; doi:10.1098/rsif.2016.0606)
Supplement: Supplementary_fixed.pdf [file rsif20160606supp1.pdf]

Supplementary Materials

Zhipeng Wang[1], Davit A Potoyan[1], and Peter G Wolynes[1]

[1] *Center for Theoretical Biological Physics, Department of Chemistry and Department of Physics and Astronomy, Rice University, Houston TX 77005*

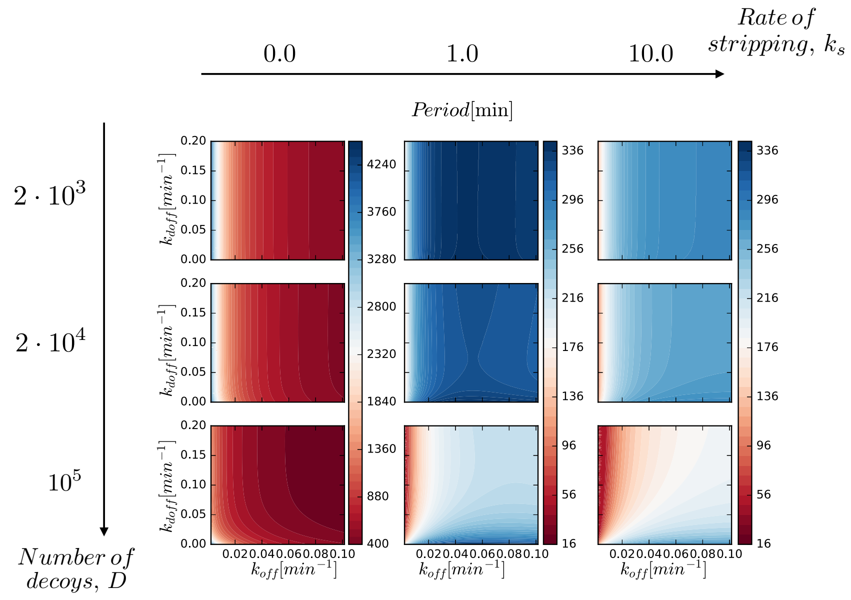

Figure 1: Period of nuclear $NF\kappa B$ oscillations as a function of dissociation time-scales from decoys k_{doff} and gene promoter k_{off} under regimes set by different decoy number (columns) and molecular striping (rows).

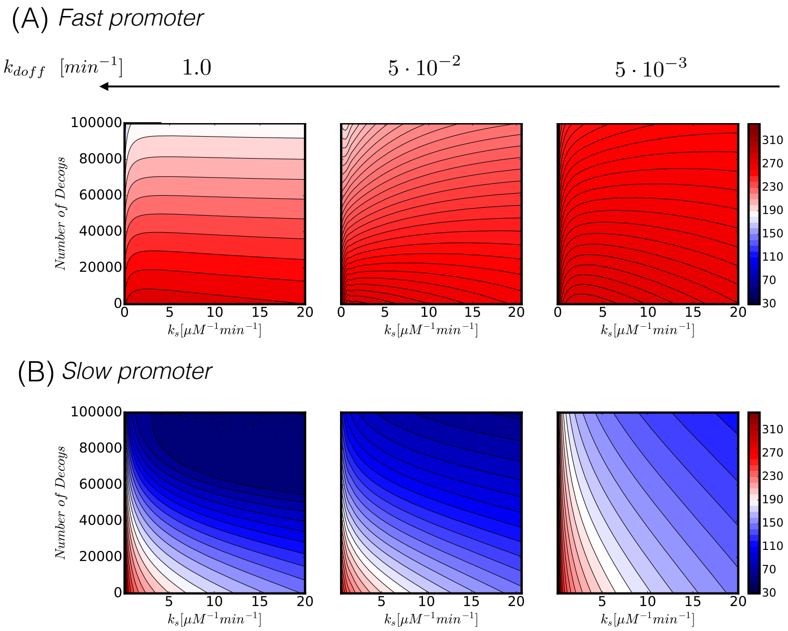

Figure 2: Period of the nuclear $NF\kappa B$ oscillations plotted as a function of number of decoys and molecular stripping rate for the regimes of fast (A) and slow (B) $I\kappa B$ promoter state change

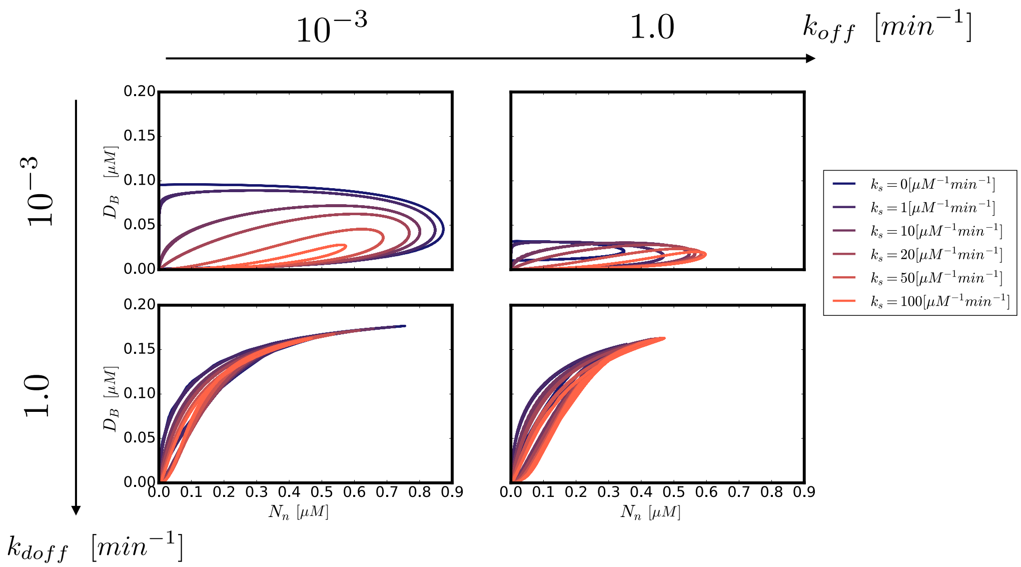

Figure 3: Limit cycles in the space of free nuclear $NF\kappa B$ (N_n) and decoy bound $NF\kappa B$ (D_B) plotted for different values of molecular stripping rates (colored in ascending order from blue to red) and for four different regimes specified by the combination of unbinding rates from decoy (column) and promoter sites (row). The total number of decoys is fixed at $D = 2 \cdot 10^4$

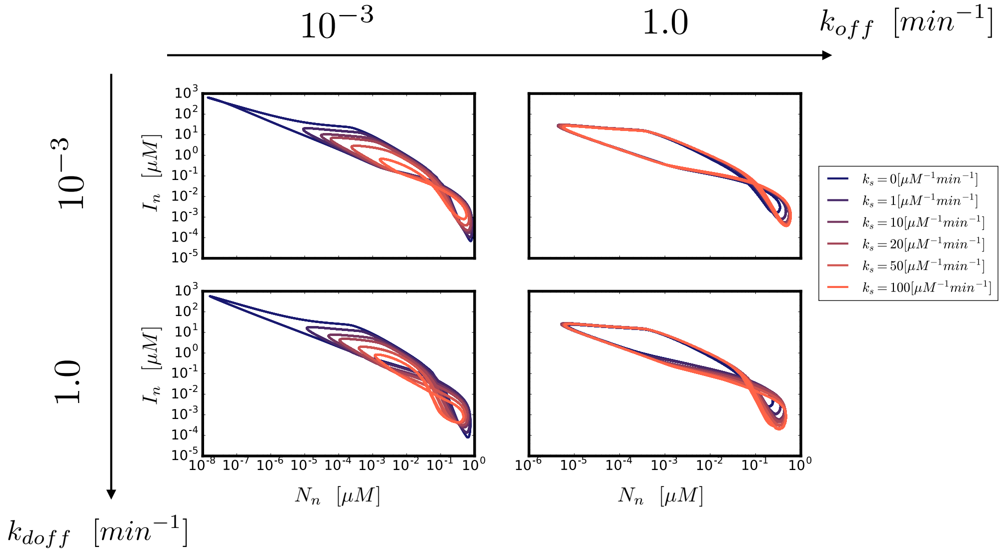

Figure 4: Limit cycles in the space of free nuclear $NF\kappa B$ (N_n) and nuclear $I\kappa B$ (I_n) plotted for different values of molecular stripping rates (colored in ascending order from blue to red) and for four different regimes specified by the combination of unbinding rates from decoy (column) and promoter sites (row). The total number of decoys is fixed at $D = 2 \cdot 10^4$

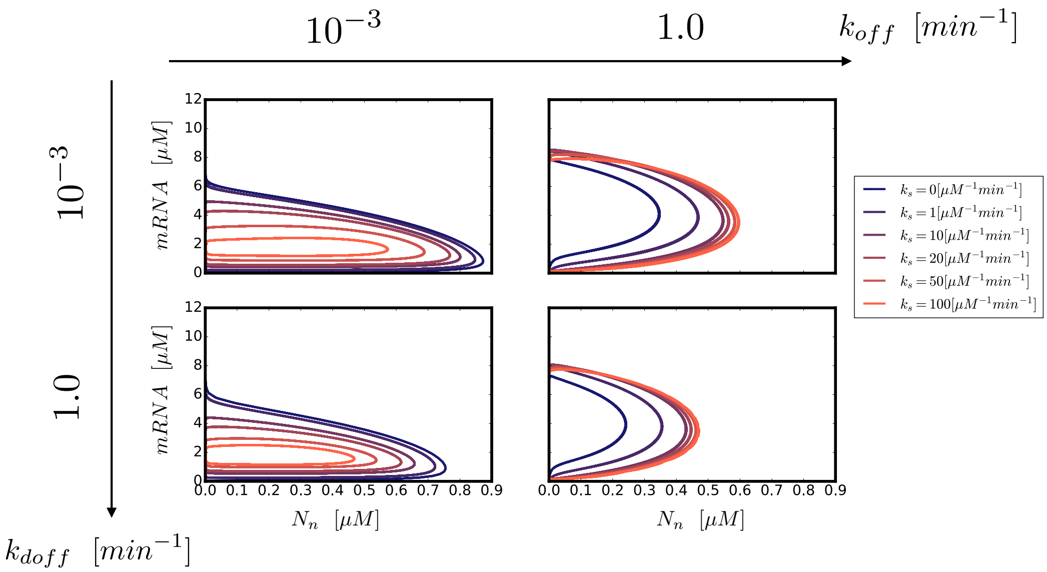

Figure 5: Limit cycles in the space of nuclear $NF\kappa B$ (N_n) and $mRNA$ (I_m) plotted for different values of molecular stripping rates (colored in ascending order from blue to red) and for four different regimes specified by the combination of unbinding rates from decoy (column) and promoter sites (row). The total number of decoys is fixed at $D = 2 \cdot 10^4$

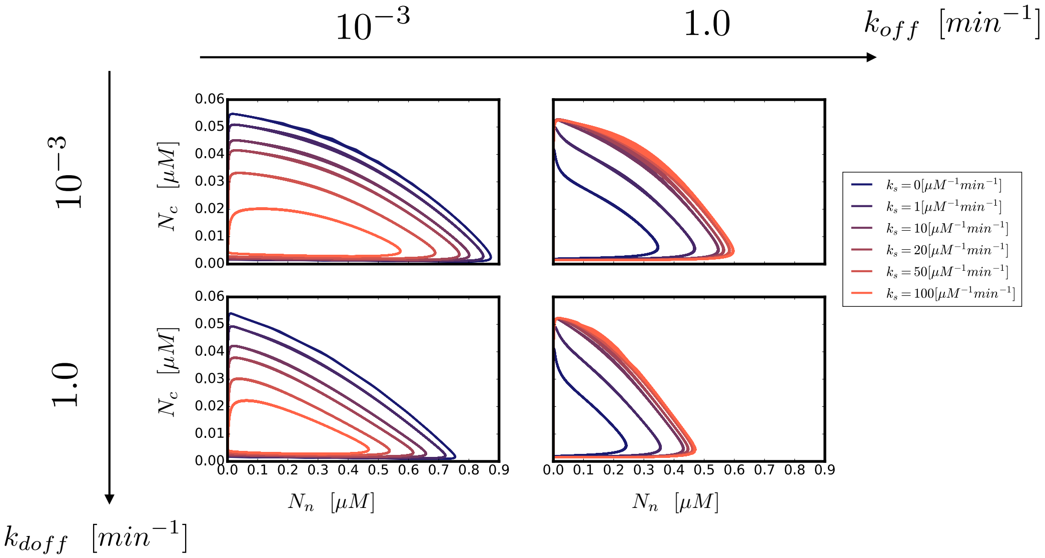

Figure 6: Limit cycles in the space of nuclear $NF\kappa B$ (N_n) and cytoplasmic $NF\kappa B$ (N_c) plotted for different values of molecular stripping rates (colored in ascending order from blue to red) and for four different regimes specified by the combination of unbinding rates from decoy (column) and promoter sites (row). The total number of decoys is fixed at $D = 2 \cdot 10^4$
